# Anæsthetics

**Published:** 1883-10

**Authors:** Levi C. Lane

**Affiliations:** Professor of Surgery


### Anesthetics.

[The Tenth and concluding one of the Lane Lectures of 1883, Delivered May 14th, at Cooper Medical College.]

BY LEVI C. LANE, M.D., PROFESSOR OF SURGERY.

In the economy of animal life nature has given two functions, viz., *Sensation* and *Motion*. In the archetype of the animal's tracing, like the leading lines these stand boldly in front and tell the observer: Here is animated life! In Nature's logic these are the major and minor premises to which some living thing bears the relation of conclusion. Each of these, if not so familiar to us, would awaken in our minds intense admiration; but to touch and to feel have been seen so often by the mental eye that, like the sun, the star, or the cloud, they have long ago dulled the sensibility, so that though they rank actually among the most deeply veiled mysteries of our being, we never think of or marvel at them.

In the background of Life's picture are other less distinctive lines or marks, which are less characteristic inasmuch as they are shared equally by Plants and Animals: these are the nutritive, the instruments of which in the plant are rootlets and leaves, whilst in the animal the rootlets of nutrition spring from the mucous surfaces of the stomach and intestines. The plant, in consequence of its nearly complete want of locomotion, is compelled to send out its radicles in quest of food in its immediate vicinity, thus differing from the animal that roams hither and thither in search of nutriment.

The line in the sketch representing motion, if generalization be strained somewhat, may be claimed by the plant, since some species do actually move their leaf-stems. This motion however, though we do admire it, is but a counterfeit and a mockery when compared with that movement "so express and admirable" possessed by the animal. The shrinking, retiring bow with which the sensitive plant droops its leaves when jostled or touched is but a shadow when compared with true animal movement. Still there is enough of it to make an important breach in the wall that the naturalist would fain raise between animal and vegetable existence. Nature loves freedom, and scorns to abide in the narrow inclosures to which Science would consign her; for though the containing wall be reared with faultless care, with the strength of adamant and the hardness of diamond, and the supposed captive be bound with iron fetters, yet Nature, Proteus-like, eludes the guards and finds a door of escape. On this account half the occupation of Science is in making new deductive guesses and shifting her would-be enduring landmarks. Though the handiwork of Nature is usually marked by infinite simplicity, yet in doing her work she exercises so much quiet prudence that to detect a single one of her footsteps Science must often take countless steps. Science, to encompass a minimum tract in the domain of Nature, often must needs use a chain each link of which represents a generation of men. Kepler, to discover the ellipse through which our Earth annually moves, must guess a thousand times, and literally cover and re-cover the face of the heavens with diagrams. Again and again did he draw from the urn of knowledge ere he secured those combination-keys which unlocked the gate of the skies and gave mankind the priceless jewels which hitherto had lain concealed there.

The animal sphere, less fortunate than the celestial one, yet awaits its Kepler. Diagram after diagram has been traced upon and again discarded from the face of the former. Bold hands have vainly essayed to draw from the sacred urn the key which may unlock the Book of Life; and links must yet be added to the chain that may successfully sound the deep sea of animal existence.

Near the close of the first third of this century great advancement was made in this department of knowledge; and perhaps it would not be presumption to say that one of its leading keys was found by Sir Charles Bell when he discovered and announced to the world that from the front half of the spinal cord of the Vertebrated animal nerve-cords spring which preside over motion, while from the posterior half arise nerves which preside over and are concerned in feeling. I think it may not be extravagant to remark that this discovery gave to old Medicine a new world, and is in rank scarcely secondary to Harvey's discovery of the circulation of the blood. Nature in this case only confessed her secret when subjected to the rack of torture; for the discovery was made by the cry of pain from the animal questioned.

As the nerves emerge from the substance of the cord they are separated from each other by quite an interval, so that in the frog, pigeon, or any of the higher animals, when the spinal canal is laid open one can readily catch and compress the nerve. Thus by pinching the anterior root, Bell found that motion was caused in the part supplied by the nerve; or if the posterior root was injured the animal gave signs of pain; and the separate functions was still more surely proven when the root was severed, since then palsy of feeling or loss of motion ensued according as section was made of one or the other. A short distance however beyond their origin the two roots join, and the fibers of the two are inextricably intermingled. Thus motion and feeling are united in an indissoluble wedlock.

Before Bell's discovery, cases of disease where there was palsy of a portion of the body, on a large or limited scale, were enigmas admitting of no satisfactory explanation. Yet as the human mind has always been intent upon finding some kind of solution where natural laws could not be discovered, the matter was transferred to the domain of the mysterious and supernatural. And where credulous superstition once placed its seal it required a strong, knowing and fearless hand to open. In the case before us, it was long ere such a hand appeared. And during the time when humanity was awaiting the appearance of the scientific evangel, there is offered to our eyes one of the saddest phases in the annals of humanity. For when History rolls back the stone from the sepulchre in which lie entombed the 15th and 16th centuries, we hear issuing thence no more pitiful wail, no more touching cry, than from those who fell victims to the accusation of sorcery.

In the records of Demonopathy which have come down to us, we find that the commonest form of possession was that in which the subject had lost the sense of feeling or the power of motion in some portion of his body. This was one of the favorite modes in which the devil was believed to torment the human body; and the half-witted victim of such ailment too often found some poor wight through whose intermediation Satan had accomplished his mischief. At this unfortunate era of our race the belief in demonopathy seems to have been nearly universal. The civil magistrate appears to have been even more orthodox than the ecclesiastic so that when the accused were transferred to the former for trial, conviction as a rule followed accusation, and death by burning was the closing scene of this awful drama. The annals of sorcery depict many such cases; and one civil judge, thinking to commend himself to posterity for his vigorous efforts at extermination of the evil, boasted that he had sent more than six hundred of such malefactors to the stake.

And the persons thus accused and condemned were not wholly the poor and the friendless. One Louis Gaufridi, a highly learned and influential priest, was burned to death in France, upon the accusation of two possessed girls. Several nuns were also executed for similarly alleged offences. Even the great Kepler had to interrupt his studies and throw himself between the law and his own mother, who was charged with practicing sorcery. Though reprieved, the wretched woman was shown in her dungeon the red-hot pincers and the rack which had been prepared for her punishment.

In the history of humanity one finds many pages darkened with these abhorrent recitals; pages which he would fain destroy; yet, on after thought, they should be preserved, as they serve as earnest rebukes to those who claim that the world is not becoming both wiser and better. Two centuries ago, insanity, St. Vitus' dance, hysteria, and local insensibility were firmly believed to be the work of demons induced by human intermediation. But before the light of intelligent Medicine the lurid glare of superstition has slowly faded away, so that to-day it would be hard to find a mind so benighted as to believe in demoniacal possession. Bell's great discovery has shown us how local injury in the nerve centers can deprive a separate portion of the body of the power of feeling. And this great truth was brought to us through the aid of vivisection; and without vivisection it is fair to state that Bell's discovery could not have been made. Yet governments are besieged by modern sentimentalism to intervene and close up this valuable route, in fact almost the only one that leads to yet unknown verities in the sphere of animal life. Governments do not hesitate to sacrifice armies of men—mutilating them in body and limb, and killing them often in the most brutal manner—in defence of some strip of soil or some commercial right; yet unthinking pity and narrow benevolence can behold all this cruel sacrifice in silence, and become noisy in its clamors of commiseration when life may be saved and pain spared to millions of its own kind by the destruction of a few hecatombs of the inferior animals. Pity, though one of the noblest qualities of the human heart, when unguided by the light of reason, can easily wander into extravagant excesses.

Let us next proceed to a closer consideration of the line of sensation, which holds so prominent a place in the schematic picture of the archetypal animal.

Suffering humanity in every language uses but one word to announce pain; while pleasure is told by a thousand sounds, represented by permutations of vowels and consonants, the story of pain is told in one expiring sound, *Ah!* From the Equator to the Pole this sound is known and familiar to all ears; no translator's aid is required to render its import intelligible; this knock at the outer door of every human heart is quickly recognized by the conscious soul within. Words, I have thought, may be so combined by some future genius as to have an irresistible power. Such a gift was partially possessed by Shakspeare. Should the future bring forth one having such perfect gift, it will be one whose intuitive powers will enable him to create or find words which, like *ah*, with irresistible, pent-up potency, will thoroughly voice the inmost feelings and emotions of the human heart.

To relieve *ah* of its bitter sadness, and to put to rest the noiseless agony in the aching nerve, are the destiny and mission ot Medicine. This is the purpose which, like a divine light, has illuminated every page of medical literature from the scrolls of Hippocrates down to the works of to-day. The pages of Herodotus, Thucydides, Livy, and Tacitus are emblazoned with the exploits of the death-dealing sword, while the spirit of peace breathes along the lines of Hippocrates, Galen and Celsus, as they tell us how pain may be lessened, disease cured, and life prolonged.

The generation of Homer, Plato, and Thucydides used the four syllabled word *aisthanomai* to express what we utter in the two words, *I feel.* From this Hellenic source Medicine, Logic, and Rhetoric have borrowed thought-germs and transplanted them into their respective spheres; germs which like the tiny seeds of our Sequoia have compressed into themselves boundless capabilities; for that transplanted into the field of medicine has budded into a prolific trunk, the prominent branches of which are hyperesthesia, paresthesia, and anæsthesia. The last branch plucked from the parent stem has been carried to the far west, and there replanted. What fruit it has produced we will hereafter learn.

As in the organic world physicists regard all phases of force as masked forms of motion, so I venture the theory, and predict that some day all sensation will be referred to the same head, and that pain in the future philosophy of life will be resolved into intensified motion, and that the amount of energy expended in the phenomenon of pain will be measured by an arc on the dial-plate of an æsthanomic dynamometer. The problem in medicine in which mankind has been most interested in the solution, has been to find means to clog the motion in the disordered nerve-cords—to check the speed of the wheels of life down the fatal declivity of disease and to restore the posterior sensory columns to the peaceful equilibrium of health. Or where complete restoration cannot be reached, how to reach partial relief.

As the knife of vivisection may be laid separately at the root of the trunk of motion, or of sensation, so certain substances when introduced into the blood act in a similar way. Thus medicine has learned to lay its finger on each of these functions, and so to speak, temporarily stop either of these wheels of life. In respect to motion medicine learned its first lessons from the aborigines who inhabit the region near the Orinoco in South America; a drop of a matter obtained as some say from the skin of a frog, as others think from a plant, has the wonderful property of depriving of motion an animal inoculated with it; and as the flesh of the animal thus palsied is not rendered poisonous, the Indian infects the points of his arrows with the curara and uses the same to secure his game. The peccary or wild hog wounded with such arrow quickly loses the power of his limbs and so becomes an easy prey to his pursuer. Such weapon became an individual aid to the Indian, both in chase and war.

Medical science, ever on the alert to add to its resources, was not slow to perceive the value of such an agent, and at once employed it to control the excessive contraction of the muscles in lockjaw. Prudently administered this agent has cured a number of cases of this frightful disease; and likewise persons poisoned by strychnia have been relieved by the use of the arrow poison. Large doses of chloral hydrate act in a similar way; this medicine, like some magician's wand, waved over the wild storm on the anterior nervous field of the tetanic subject, often quickly brings quiet, and speedy rescue from a painful death. The discovery of this agent was not due to the aborigines, nor was it the result of an accident or a lucky blunder; but we owe it to the keen intelligence of an experimental physiologist—Liebrich, of Berlin—who compounded and first learned the action of chloral on the lower animals. The medicines here mentioned are capable of great harm as well as of great good, so that the hand which skillfully uses them must have both daring and caution—daring, to slow the wheels of life, and caution, not to stop them. The timid hand fearing to do too much, halts too early, while the rash one may overreach the aim; for despite the boasted powers of our art, with the great dramatist we must confess that when once the vital spark has been extinguished, we know not where is the power that can this light relume.

Though great praise is due, yet it has been inadequately rendered to such men as Liebrich and others, who in the noisome fumes of the laboratory are determining by close observation and careful experiment the action of remedies upon the animal body —the frog, pigeon, hare, and dog lending their bodies for this purpose. *Inadequate*, I say, is the praise which the world renders to such men; for while every school-child is familiar with the names and exploits of Alexander, Cæsar, and Napoleon, such names as Dare, Hunter, Jenner, and Lister are hardly known. The destroyers of the race have been idolized, nay, deified, while its true benefactors have lived and died leaving but slight hold on human memory. And what is still more to the honor of these benefactors is that they labor with the foreknowledge that the field in which they toil brings but stunted laurels. The child named Alexander or Napoleon knows not the name of Jenner, whose great discovery has rescued it from permanent deformity if not from a loathsome death. And all this is owing to the truth that the precious standard of regular medicine has ever permitted but one inscription on its banner, namely, Modesty.

As the motor roots are under the control of curara and chloral, so we have a large list of medicinal agents which influence or control the posterior or sensory nerve-roots; and these are known as narcotics, anodynes, and anæsthetics. The necessities of man have incited him to keen research in this domain, hence the list of remedies known which act thus is great in number.

Every physical pleasure of man falls under the head of sensation; and as a paradox and seemingly incredible contradiction, all his physical woes lie within the same category; and hence in one circle the most antagonistic conditions exist. If some Mercator could project the great sphere of organic being, pleasure and pain would occupy the same, opposite hemispheres. To make the projection perfect, the hemispheres should so intersect as to have a tract of common territory—a species of neutral ground—where neither pleasure nor pain exists exclusively. In this neutral ground we find that most of our existence has been passed, if we confess to the truth of our individual experiences.

Some years ago, while visiting at the private residence of a wealthy gentleman of this city, I was much struck with the wondrous beauty of his place, and ventured the remark that amidst such scenery and surroundings he must be a perfectly happy man. His reply showed that though a man of trade his thoughts had passed beyond the range of mercantile matters, and that reflection and observation had led him to a due estimate of the limits of human felicity. He replied: "The conditions of human life are consistent with but few moments of happiness, and those are brief and only occur at long intervals." If mankind would adopt such a standard in their expectations of the future, and with Horace were to clip the wings of extravagant hope, there would be much curtailment in disappointment. Hope too often beckons on to silvery clouds which when reached and grasped are but empty, chilly vapor.

Human existence, however, is not even so fortunate as to have constant abode in the drowsy neutral section mentioned; its bark is often driven, or suddenly cast by accident or destiny, on the shore of pain. From the very couch where existence has its succession and continuation the cry of anguish never ceases to ascend; every industrial enterprise which contributes to human comfort and ease of life through fabrics wrought by steam and machinery, often crushes the limb or mangles the hand of the artisan; almost every surgical operation, as amputation, ligation of vessel, removal of stone, etc., is accompanied by a rude assault upon the sensory portion of the human body. To exorcise this demon Pain, in its forms so numerous indeed that

> "To count them all would need a thousand tongues,
> A throat of brass and adamantine lungs,"

did in the most remote eras of our race awaken effort and incite search for medicines which could give relief. And this was especially the case where Surgery was called upon to intervene and make wounds in the human organism. For, to cure wounds, or to pluck from the diseased organism some useless member or offending agent, wounds must be made. To do this with the least pain to the sufferer, if we turn back the pages of medical history, we find many things suggested, some of which we will now proceed to mention.

Long prior to the dawn of English civilization, Medicine as well as other sciences had made considerable progress in Egypt. In the Museum of Cairo, I saw a number of instruments obtained from the interior of the Pyramids which had evidently been employed in surgical operations; one sees knives which evidently were used in cutting for stone; also an instrument that might have been employed as forceps in midwifery. From the early medical annalists we are informed that to render the patient insensible who was to be operated on, the Egyptian physician administered opium and Indian hemp. With the same purpose, though ludicrously erroneous, the material that was used for the moxa—a species of cautery in which some vegetable substance was allowed to slowly burn on the skin—was composed of hemp.

In the third century Moa-Tho, an Oriental surgeon, also used Indian hemp to relieve pain during surgical work; and as a more effective agent against pain he also used hasheesh, an extract from hemp.

As a singular way to induce insensibility, Pliny the Second mentions a stone named memphitis, so named from the place of its origin; this stone having vinegar poured upon it and then being applied to a part of the body, such part could be cut or burned without causing pain. It is probable that the vinegar caused the escape of some gas from the stone, which benumed the part with which it came in contact. This mode of inducing local anæsthesia has its counterpart in Richardson's plan of partly freezing the part that is to be operated upon.

Dioscorides, a Greek writer, mentions the use of Atropa mandragora as a means of inducing insensibility to pain; a decoction was made of the mandragora by boiling it in wine, of which the person to be operated on drank a portion. Pliny also mentions that if one drank of this mandragora, though he were cut or stabbed or bitten by a snake, he felt no pain. And at a later period, Shakspeare in his ubiquity of knowledge alludes to the narcotic virtues of mandragora.

In the 13th century the administration of remedies for anæsthetic purposes was so prevalent that it attracted the attention of the Church. As it was then the custom to often voluntarily subject one's self to pain, it was deemed impious to attempt to escape the pains of physical ailment, and that he who did so was opposing the divine will; hence in 1287, in a Church Council, Origenes called attention to the practice of physicians who to perform operations administered some soporific which deprived the patients of their senses.

In 1298 it is recorded that Theodoric de Cervia made a compound of opium, mandragora, cicuta, lactuca, and hyoscyamus, which he placed on a sponge and allowed those to breathe upon whom he intended to operate; and when the work was concluded, to awaken the patients from the sleep that had thus been induced, he caused them to breathe the fumes of vinegar; and this failing to arouse, he placed the juice of rue or fennel in their ears.

In the celebrated School of Salerno, in the south of Italy, where medical degrees were first granted to students, one Montagna, a surgeon, gave a somnific portion to those upon whom he purposed to perform operations.

A little later than this, old Bartholin tells us that his preceptor, Marcus Aurelius Severinus, used snow to induce local anæsthesia. For this purpose snow was placed in an oblong vessel and directly applied for fifteen minutes to the part which it was intended to render insensible; the part thus chilled became benumbed and deprived of feeling; gangrene did not follow such freezing. The process of Severinus differed in its results in no wise from that of Richardson; Richardson instead of snow or ice using the cold vapor of ether to freeze the part.

In the last quarter of the 18th century, Sassard of Paris gave narcotics to lessen the pain of operations and the shock which results from the same. About the same time James Moore, of England, instead of internal remedies or the external use of cold, acted directly on the trunk of the nerve supplying feeling to the part to be operated upon. And for slight cutting on the limbs this is an expeditious and excellent means to produce insensibility; for example, to lance a felon, pressure on the median nerve at the wrist will render the work comparatively painless.

Towards the close of the last century Chemistry was greatly advanced and placed on a permanent basis by the labors of Lavoisier; his ingenious nomenclature relieved the arduous work of memory and enabled the student to quickly acquire a comprehensive view of this science. In the midst of his great discoveries Lavoisier fell a victim to the French Revolution. This great man was apprehended on some trifling charges which were never proven, and despite the efforts of his friends was condemned to death. Conscious of his innocence, and firmly believing that in the counsels of supreme destiny there yet remained work for him to do, he seems never to have fully realized his danger until informed that the "Republic had no need of Chemistry." Yet Chemistry could not be guillotined, and through the impulse given to it by Lavoisier it was rapidly developed, and furnished Medicine with new means of combatting disease.

The new agents, oxygen and nitrogen and their compounds, were conceived to possess medicinal properties, and trials of them were made by various persons, among whom may be mentioned Priestly and Humphrey Davy. Early in this century Beddoes founded his Pneumatic Institute at Bristol for the treatment of disease by inhalation, The establishment was for a time under the charge of Humphrey Davy, then a young man, who made various experiments with nitrous oxide—familiarly called laughing gas—and discovered its anæsthetic properties in the relief of toothache and headache. Guided by his observations and experiments, in 1800 Davy wrote the following remarkable words: "*As this gas seems capable of destroying physical pain, it may probably be used with advantage in surgical operations.*" These words were manifestly written without thought of their scope and significance. Prophetic words which were to be fulfilled forty-six years afterwards, but of the meaning of which the utterer himself was unconscious! The germ however was planted which was destined to grow, blossom, and ripen into the most precious fruit that has ever been given to humanity.

Near the same period there is repeated mention made of the use of ether by inhalation, both on the Continent and in England. In Nysten's dictionary, published in 1815, there is a description of an apparatus constructed for the administration of ether by inhalation. Faraday states that a mixture of air and the vapor of ether when inhaled produced the same effects as laughing gas. As one with the light of present knowledge reads these facts he is astonished that the discovery of anæsthetics was not then made. The world in its thoughtless march, like a footman meditating on trifles he knows not of, moved on in its unthinking mood, taking no note of objects which lay plainly in its field of vision; for the world, like the footman, is only roused to consciousness when it stumbles or is painfully jostled. Hence for a period yet the inhalation of ether and nitrous oxide remained as toys with which students and professors amused themselves in the laboratory. This golden chain, whose links we have followed from the remote Orient along the Nile, through classic Greece, Rome, and the land of the Western Nations, was actually completed in the commencement of our century, yet no one knew how to use it.

As in the chase the pursuer is often misled and diverted from his object by the artifices and detours of the fleeing prey, so in the development of the arts of human culture some precious discovery seems, when actually in reach, to have playfully eluded the grasp of one or more generations. This was the case in the history of anæsthetics; for instead of seizing hold and making use of what was in plain sight at this point, some of the leaders in the medical profession strayed away into the region of the occult and the mystic, and becoming lost in the fogs and morasses of Mesmerism, mistook the will-o'-the-wisp there for the true anæsthetic; and thus a half generation was lost in trying to catch this intangible vagary. And we are told that in 1829 Cloquet amputated the breast of a woman who had been mesmerized; likewise in England one Dr. Ward is said to have amputated a thigh of a person who had been put to sleep by the mystic passes of a mesmerizer.

In the Northwestern portions of London the strolling traveler's eye is attracted to a sepulchral monument of no mean pretention, and evidently so placed as to render it especially conspicuous to the passer-by. From the inscription one learns that it is the grave of Perkins, the discoverer of the pretended fact that when metallic rods or *tractors*, as Perkins called them, are brought in contact with the body of one suffering, his pains are quickly dispelled. For more than two generations the tractors have been consigned to the rubbish closet, and oblivion has about established its claims to the inventor's name; and the pompous phrase in which his immortality is heralded on his tombstone has nearly been blotted out by the smoke and rain of London.

Nearly cotemporaneous with the tractors was the discovery of Hypnotism by Dr. Braid. This singular condition of the human body is induced when the subject is caused to look steadfastly at a bright metallic point for some minutes, whereupon the senses are caused to fall asleep, and the person thus being rendered insensible may have surgical operations performed upon him without his knowledge. In Paris, Broca, Follin and others thus performed surgical operations. Yet it was soon found that the number of persons who could be rendered insensible by Hypnotism was so limited that as a means of anæsthesia it could not be adopted for general surgical practice; though in the East Indies where the population seems to be especially susceptible to hypnotic agency, one English surgeon operated thus in more than one hundred cases. Hence the hypnotic meteor has faded to a penumbral shadow destined soon to undergo total eclipse.

The dream-land of Hypnotism was the last stage through which the search for an anæsthetic passed before its ultimate triumphant realization. In the Winter Palace of St. Petersburg one sees a remarkable picture representing a vision of Peter the Great during the night which preceded the battle of Pultowa, in which the future of Russia was to be decided, whether its destinies hereafter should be wielded by Sclavonic or Scandinavian ruler. Above the sleeping chieftain, in the shadowy clouds of night, one sees the tutelary genii of the North hovering over his head, holding wreaths of victory in their hands. So in the gloomy sky of Hypnotism the Spirit of Discovery was hovering over expectant humanity holding in his hand a chaplet of victory that far outweighs that won by the hero of Pultowa.

But, as if too great glory for one man, the honor of this discovery must be given to four men.

In 1842–3, W. C. Long, of Athens, Georgia, used ether as an anæsthetic for surgical purposes. The claims of Long have been defended and satisfactorily established by his friend and fellow-countryman Dr. Marion Sims; but the failure of Long to publicly announce his observations deprives him of much of the credit which otherwise would have been awarded to him. For, in the Court of Science, prior publication confirms and establishes claim to discovery.

Next, in 1844 Horace Wells, a dentist of Hartford, Connecticut, having learned from one Colton the benumbing effect of nitrous oxide decided to test it on himself and actually had a tooth thus extracted, and experiencing no pain he next tried it in some twelve or fifteen cases of dental extraction. Wells becoming thus satisfied of its efficacy as an anæsthetic, went to Boston, Mass., and offered to demonstrate the action of nitrous oxide in one of the hospitals. Before a class of medical students (by no means a proper place to test so delicate an experiment) Wells attempted to anæsthetize a patient, but utterly failed to do so; and being intensely mortified and overburdened with despair he returned home, and soon afterwards ended his existence by suicide. Time, however, has done justice to his memory, in the fact that his native city has erected a monument to his memory and granted a pension to his widow.

In 1846 Morton and Jackson, of Boston, appear on the anæsthetic arena and carry off the long sought for prize. There is strong evidence that these men were present and witnessed the failure of poor Wells, and that had they—at least one of them (Morton)—been in Wells' place, success instead of failure would have crowned the undertaking. Jackson was an experimental chemist, and from his trials with sulphuric ether he became satisfied that it might be used to render subjects insensible during surgical operations. Morton, at Jackson's suggestion, gave ether to a patient and successfully removed a tooth without causing any pain. Morton (it is said, at Jackson's suggestion) next imparted a knowledge of his experiments and their results to Dr. Warren, the famous surgeon to the Massachusetts General Hospital.

It required a bold spirit, a mind unshackled by the fetters with which routine shackles the ordinary professional man, and a nature ready to assume great responsibility, to so far forsake the beaten paths of surgery as to operate upon a patient in a state of unconsciousness. Even after the anæsthetic use of ether had been demonstrated by many trials to be free from danger, many of the leading men in medicine were utterly repugnant to its employment. The great Dieffenbach, in my opinion the greatest surgeon of that time—or, if he be given due credit for all his original surgical procedures, the greatest of all times—has left on record a strange transcript of his feelings when in the presence of a patient rendered insensible by an anæsthetic: "How isolated and desolate does the surgeon find himself in the presence of a patient whom he has so robbed of his senses that he can respond to no question and can receive no word of cheer from the operator! How melancholy is the situation of both patient and operator when the chain of sympathy which should bind the two together is broken! How irrational a procedure to first induce in the patient a dangerous disease in order to remove another!" From this paraphrased compend of Dieffenbach's picture of his feelings when in the presence of a patient under the influence of an anæsthetic, one infers that so far from his having had the courage to initiate such an innovation it was evidently one of the regrets of the old hero in surgery that he had lived to see such a departure from established custom. Hence it was greatly to the credit of Dr. Warren, and must ever remain as his chief laurel, that he was the first to have the daring to perform a grave surgical operation, viz., the removal of a cervical tumor from a patient under the influence of ether. In doing this, Warren showed that he was not a degenerate but a worthy descendant of that ancestral lineage that had poured out its life-blood on the altar of patriotism.

This operation was done on the 11th of October, 1846, and was the successful and triumphant inauguration of the use of anæsthetics. This day, marked in golden letters, ushered in a new era in the annals of medicine.

In our history of anæsthetics one thing has been untouched, or but too lightly touched upon, viz., the work which the dental profession has done in the solution of this great problem. The exquisite torture, though but of a moment's duration, which attends the pulling of a tooth, has long appealed to the ingenuity of the dentist and stimulated him to find some way of doing the work with less pain. Whether modesty or what not has deterred the dentists from preferring their claims, I know not, but to them the credit is certainly due of having made the first successful essays in the anæsthetical use of nitrous oxide and sulphuric ether.

On the 18th of October another surgical operation was successfully done by Hayward, the patient being under the influence of ether. And early in November a cancerous breast was removed and a leg amputated by the aid of a new agent. Thenceforward news of the great discovery was carried as rapidly as steam could bear it to all regions of the earth where intelligent Medicine exists. So that early in 1847 reports came of operations performed under ether by most of the leading surgeons of Europe.

As the administration and action of ether were found to be accompanied by numerous inconveniences, hence the inquiry early arose whether some better anæsthetic could not be found. The most earnest worker in this direction was Sir James Simpson of Edinburgh. After trying numerous agents Simpson gave his decided preference to chloroform, and published his observations made upon some eighty cases in which chloroform had been given. Under the impulse of the high authority of Simpson, chloroform become at once the rival of ether as a means of inducing anæsthesia. In our country, Boston, as the birth-place of ether, with the fondness of a parent has consistently given her preference to ether; while in New York and most other American cities, as far as my observation has extended, the preference is given to chloroform. In France the School of Lyons is a determined partisan for ether, while Paris adheres to her early preference for chloroform. Vienna has compomised the matter by using a mixture of the two. In my visit not long ago to London, I noticed that ether was rapidly superceding chloroform. In Berlin and Munich chloroform occupies undisputed sway. Observations now reaching over a third of a century have shown that neither is free from danger; and the Pitrequin of Lyons, the warmest champion ether ever had, was in too much haste when in 1866 he proclaimed that ether never kills; for in less than two years afterwards Gavet and Laroyenne reported six deaths from the use of ether. So too, in Paris, Sebillot was premature in announcing that "pure chloroform properly administered never destroys the patient;" for some years afterwards when a number of fatal cases from chloroform had been reported, Sebillot wrote: "Chloroform is not without danger; still, one can shelter himself from accidents through ability, experience, and attention."

Careful study of the mode of action of each of these agents, as well as a knowledge of the symptons which announce peril, place it in the power of the learned and watchful physician to greatly reduce the danger. In steering through this perilous sea the administerer of the anæsthetic should ever have three beacon lights in his eye—the pupil of the eye, the breathing, and the pulse; and should his eye stray from these and become fascinated with the surgeon's work (a thing that too often occurs) the patient's life falls into immediate jeopardy; for the widening of the pupil that has once been contracted indicates the opening of the door through which death will soon enter; the irregular or halting breath is the footstep of coming danger; while the sudden cessation of the pulse announces, even though breathing continues, that Death has entered and holds possession of Life's innermost citadel.

From a large list of administrations reaching over many thousands of cases, both in America and Great Britain, the average risk to life has been pretty accurately determined. It amounts to about one death in 2800 administrations. When the administration of anæsthetics becomes an isolated profession, and shall become the business of men who shall do nothing else (an example of which is the celebrated Dr. Clover in London), then it is probable that the mortality will fall much under that above given. An inexcusable procedure is where the surgeon attemps to perform the double office of administerer of the anæsthetic and operator at the same time; likewise where after he has anæsthetized the patient, he commits the further administration to unskilful hands; in each case the chances of the patient dying are greatly increased. That physicians and surgeons should so far forget the rules of their art as to take such risks, springs from the fact that when one has often witnessed the administration he gradually forgets the attendant danger. Yet such danger is always near, and since like an insidious enemy it is apt to appear when least expected, hence the watchman at this important post of professional duty should never sleep lest death should be the punishment.

On the other hand in the use of anæsthetics enough should be given to produce insensibility, for unless the patient be put fully to sleep he will suffer more than if he had really taken none at all. The timidity of the administerer often causes him to commit such a fault. And it is due to this that in professional experience we often meet patients who tell us that ether or chloroform will have no action upon them. This is an error, and as it causes considerable evil it should be strenuously combated, since there is no living person that is not susceptible to the power of anæsthetics, though there are some on whom the administration might be improper.

During the war of the Rebellion 23,260 persons were operated upon while under the influence of anæsthetics; chloroform was used in nearly two-thirds of the cases.

But let us refer again to Jackson and Morton, to whom as we have seen was mainly due the final discovery of anæsthetics. The idea of realizing a great fortune from the discovery early suggested itself to both of them, and especially to Morton, who seems to have been endowed with great energy and a keen desire of gain. It was first proposed to take out a patent and thus control the sale of the anæsthetic agent, the exact nature of which for a time was held as a secret. Jackson, however, more desirous of glory than money, early retired from the patent enterprise and hastened across the Atlantic, where he claimed the sole credit of the discovery. In Paris he was awarded the honors due to a great discoverer, and received from the French Government the decoration of the Legion of Honor. Meanwhile an implacable enmity and a rancorous dispute arose between Jackson and Morton, in regard to the discovery. In the House of Representatives of the United States, where Morton sought to obtain a pension as some reward for the discovery, a committee appointed to examine and report upon the subject assigned the right of priority to Morton, yet he failed to get a pension. Boston, certainly very competent to judge of the question, though the decision of the Massachusetts Medical Society also assigned the claim of priority to Morton. A bitter altercation continued between the two for years, until finally Jackson's intellect toppled over and he became the subject of a mild form of insanity, from which he never rallied, but after some years of pleasant delusion his life ended in a house of the insane. Morton, after years of fruitless struggle for compensation, finally wearied, worn, and half demented from excessive mental strain and constant disappointment, was seized with wild delirium in one of the public parks of New York and rushed to a reservoir of water and tried to drown himself. Though rescued, he soon afterwards died. Thus an intensely sad fatality attended the lives of Wells, Morton, and Jackson, the three leading actors in the discovery of anæsthetics.

It is strange that destiny should have presented her most bitter cup to the lips of three of the greatest benefactors of mankind. Though so bitter, yet it is probable that few men would decline it could they see, as certainly two of these discoverers must have seen, the chaplet of immortality with which the cup was enwreathed—a wreath woven of olive leaves whose immaculate verdure is tarnished by none of the blood-drops which stain the warrior's laurel. If we unfold the latter, there are found hidden amidst its leaves sighs, pain, sorrow, and death; while the olive chaplet of the former is sullied by no tears, and there will ever rise from it the perfume of endless good.

In conclusion, we have followed the search for the Anæsthetic from the primitive dawn of medical science, through various races and languages, down to its final discovery. And this discovery, proud am I to announce it, has been made in our own country and is destined to be one of its lasting glories. One of the labors of Hercules has really been fulfilled, since the Genius of Medical Science has sent her messenger to the Hesperian Isles of the Far West and there discovered and brought back the golden apples destined to bless all future generations.

